# Structure and Adsorption Performance of Cationic *Entermorpha* *prolifera* Polysaccharide-Based Hydrogel for Typical Pollutants: Methylene Blue, Cefuroxime, and Cr (VI)

**DOI:** 10.3390/gels8090546

**Published:** 2022-08-29

**Authors:** Xiaolei Ma, Duomo Duan, Jinbin Chen, Baolong Xie

**Affiliations:** 1 The Institute of Seawater Desalination and Multipurpose Utilization, SOA, Tianjin 300192, China; 2 Tianjin Rehabilitation Center, The PLA Joint Logistic Support Force, Tianjin 300191, China

**Keywords:** cationic enteromorpha polysaccharide, hydrogel, structural, adsorption

## Abstract

Hydrogels with polysaccharides as high polymer substrates have surprising advantages in wastewater treatment with complex components. Therefore, in this study, polysaccharides named EPS were extracted from *Enteromorpha prolifera*, a coastal pollutant with a wide range of sources, and cationic modification was performed to obtain CAEPS, the hydrogel with a double network structure was prepared based on EPS and CAEPS. Meanwhile, the structural characteristic of EPS and CAEPS-based hydrogel were identified by HPLC, AFM, FT-IR, TGA, SEM-EDS, Pore size distribution, and WCA, which showed that the porosity, apparent (skeletal) density, and hydrophilicity of CAEPS-hydrogels. We used nonlinear isotherms to uncover the adsorption mechanism of hydrogel applied to the water environment containing three typical pollutants (Methylene blue, Cefuroxime, and Cr (VI)). The results showed that the adsorption isotherm of the two hydrogels fit the Langmuir isotherm model, which indicated the monolayer adsorption of the pollution factor onto EPS- and CAEPS-hydrogels. The maximum adsorption capacities of CAEPS-hydrogels were higher than EPS-hydrogels, which indicated the microstructure and adsorption performance of the CAEPS-hydrogel are strengthened.

## 1. Introduction

With the rapid development of industrialization and urbanization, humans are facing the unprecedented challenge of water shortage [1]. Untreated wastewater from paper, printing, batteries, textiles, leather tanning, pharmaceuticals, and food processing produces many organic pollutants (phenols, pharmaceuticals, dyes, and oils) and inorganic pollutants (nutrients and heavy metal ions). The pollution is seriously damaging the earth’s water environment, and if left untreated water supplies will not meet the end of consumption in the future [2]. Meanwhile, these pollutants could be carcinogenic and mutagenic, causing dysfunction and damage to multiple organs and systems even at concentrations of about per million level [3]. In addition, some hazardous contaminants are resistant to biodegradation and remain in the ecosystem even after a long period and multiple circulations [4]. Therefore, it is of utmost importance to separate and remove toxic inorganic and organic contaminants from wastewater. Among various wastewater remediation technologies [5], adsorption based on chemical and physical interaction surface phase transfer is one of the most widely used strategies due to its low cost, easy treatment, and high efficiency [6]. Adsorbents such as activated carbon, metal oxides, clays, and polysaccharide-based hydrogels and their composites have been used to adsorb pigments, antibiotics, and heavy metal ions [7,8,9,10,11,12]. In general, raw material selection for adsorption materials is based on the principles of natural origin, availability, economic feasibility, and modifiability. Meanwhile, the porosity of adsorbents is one of the main factors determining the adsorption capacity of adsorbents [13,14]. The adsorbent with a porous structure has a large surface area, which can provide enough binding sites for pollution molecules [15,16]. Therefore, the preparation of porous structure gels by polymers has attracted extensive attention; therein, polysaccharides have been considered to be excellent materials for the synthesis of porous hydrogels as the hydrophilic natural polymers [17,18,19].

Polysaccharides, a wide range of carbohydrate polymers derived from natural sources, are widely used as raw materials for sorbents due to their excellent adsorption properties, reproducibility, biodegradability, biocompatibility, and ease of modification [20,21,22]. In the past years, more and more studies have focused on the preparation, adsorption enhancement and adsorption performance of polysaccharide-based hydrogels. Heavy metals were used as pollutants in some studies. Facchi et al. [23] fabricated a carbohydrate hydrogel composite to eliminate Pb^2+^ from wastewater, which was composed of chitosan/alginate, Fe_3_O_4_ and SiO_2_. Song et al. [24] fabricated a carboxymethyl cellulose (CMC)/polyethyleneimine (PEI) hydrogel for Cr^6+^ removal. Zeng et al. [25] prepared a 1,2-bis(2,3-epoxypropoxy)-ethane (BEPE)-pullulan/polydopamine hydrogel for Cu^2+^, Co^2+^ and Ni^2+^ removal using a one-pot method. Lai et al. [25] prepared a polysaccharide-based hydrogel (GXT) for removal of Pb^2+^ using an ice-templating technique. In addition, there are studies on dyes as target pollutants. Sethi et al. [26] synthesized a carboxymethyl cellulose/gelatin- silver nanoparticles hydrogel for removal of rhodamine B and congo red dyes. Mittal et al. [27] successfully synthesized a xanthan gum based-hydrogel composed with graphene oxide nanosheets. In addition, a class of studies focused on the adsorption performance of hydrogels for antibiotics and nutrients. Li et al. [28] prepared alginate-based hydrogel for the removal of ciprofloxacin hydrochloride. Sun et al. [29] designed a cellulose adsorbent to remove antibiotics. Zhang et al. [30] prepared a PAA/CTS/BC-hydrogel consisted based on (acrylic acid)-grafted chitosan and biochar for removal of ammonium. These studies have been interpreted from the perspective of different pollutants polysaccharide-based hydrogels could be used as potential adsorbents at an industrial scale for wastewater remediation. Substantially, according to reports it is had been proved that polysaccharide hydrogels have unprecedented advantages as potential adsorbents at an industrial scale for wastewater remediation.

In the past 14 years, the large-scale outbreak of *Enteromorpha* seriously affected the environment of coastal cities in northern China. A large amount of washed ashore *Entermorpha prolifera* consumed lots of manpower and material resources for salvage [31], transshipment, and centralized treatment. The accumulation and decay of *Enteromorpha prolifera* that has not been dealt with in time has seriously affected the living environment of coastal residents and the development of the coastal breeding industry [32]. *Enteromorpha* as a non-vascular aquatic organism in protozoa contained plenty of polysaccharides [33] and its molecular chain has abundant reactive groups, which are modified easily and also can interact with organic molecules and inorganic ions in various ways [32]. The hydrogels based on *Enteromorpha* polysaccharides were applied to the treatment of water pollution, which not only takes advantage of its natural characteristics (rich functional groups [34,35,36], wide sources, low cost, and biodegradation) but also provides a new practical perspective for waste treatment and resource utilization of *Enteromorpha* with important environmental and economic significance.

At present, there are few studies on the structure analysis and adsorption characteristics of cationic *Enteromorpha* polysaccharide-based hydrogels. In this study, the simple extracted *Enteromorpha* polysaccharides were cationic modified and prepared for double-network hydrogel by two-step crosslinking method, and its adsorption properties for three typical pollutants [3,37,38], MB, Cefuroxim, and Cr (VI), were studied. The research results of cationic *Entermorpha prolifera* polysaccharides as gel framework to prepare porous adsorption materials not only provide low-cost and biocompatible raw materials for hydrogel preparation but also provide the research thinking for resource utilization of *Entermorpha proliferate*, which is great significance for the preparation of green materials for wastewater treatment.

## 2. Results and Discussion

### 2.1. Characterization of EPS and CAEPS

#### 2.1.1. Monosaccharide Composition of EPS

**Figure 1:**
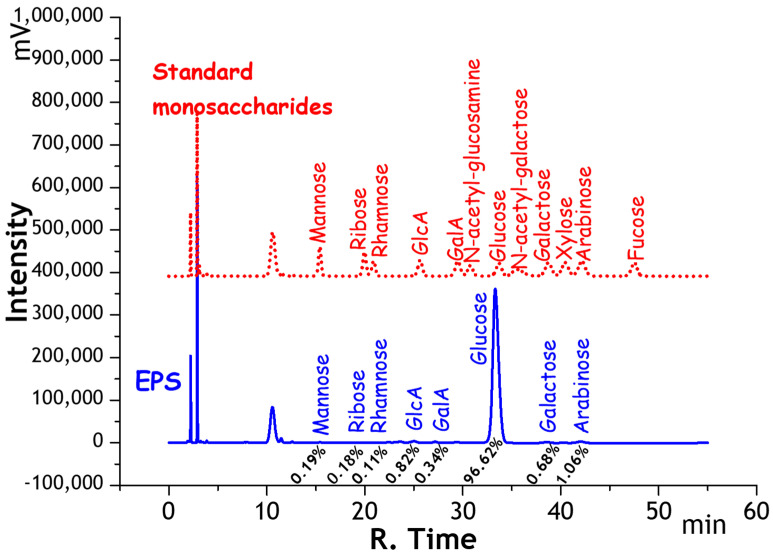
HPLC of standard monosaccharide derivative mixture and hydrolyzed derivative of EPS. Note: The samples were analyzed by the HPLC equipped with an Xtimate C18 column (4.6 mm × 200 mm × 5 µm) and a UVD at 250 nm determined wavelength.

The monosaccharide composition of EPS was measured by HPLC. Figure 1 shows that EPS was a complex heteropolysaccharide that was composed of Mannose, Ribose, Rhamnose, Glucuronic acid (GlcA), Galacturonic acid (GalA), Glucose, Galactose, and Arabinose, which was similar to the previous study by Jie Xu [35]. The monosaccharide with the largest content was glucose, whose peak area accounts for 96.64%; uronic acid accounted for 1.16%, which was composed of GlcA and GalA at 0.82% and 0.34%; and neutral sugar peak area accounts for 98.94%, which were also confirmed in a previous study [35], where the component PHPE-2 from *Enteromorpha prolifera* with high antioxidant activity contained a considerable amount of uronic acid at 21.8%. The proportion of uronic acid indicated that the EPS had a small number of carboxyl groups which would provide anion adsorption sites for the cationic pollutant.

#### 2.1.2. AFM

**Figure 2:**
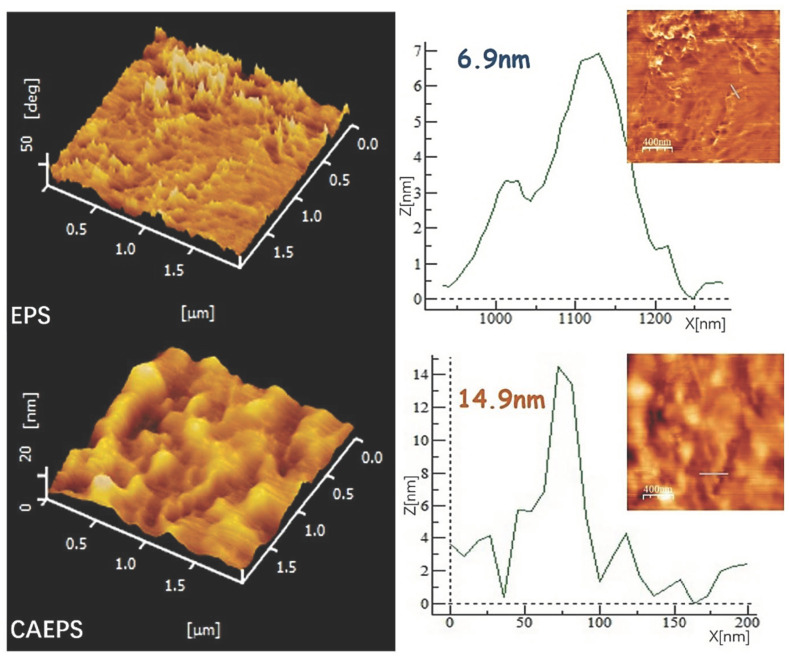
AFM image of EPS and CAEPS on micas. Note: EPS and CAEPS were prepared into a solution with a concentration of 0.1 µg mL^−1^ for measurement.

In this research, the chain morphology of EPS and CAEPS was analyzed by an atomic force microscope at different magnifications, and the altitude difference of the chain structure was measured. It can be seen from Figure 2 that EPS and CAEPS were not fully stretched in water, and there are fewer polysaccharide molecules on the mica sheet in the form of a single molecular chain structure, which showed a chain structure of agglomerates as highlight points. At the same magnification, the height of the similar chain structure of EPS was 14.9 nm, higher than that of CAEPS. This indicated that, compared with EPS, the CAEPS main chain stretched more fully, which may be related to the cationic group. Significantly, the samples on the detection of AFM, FT-IR, TGA, SEM, and pore size distribution were all xerogels obtained after freeze drying.

#### 2.1.3. FT-IR

**Figure 3:**
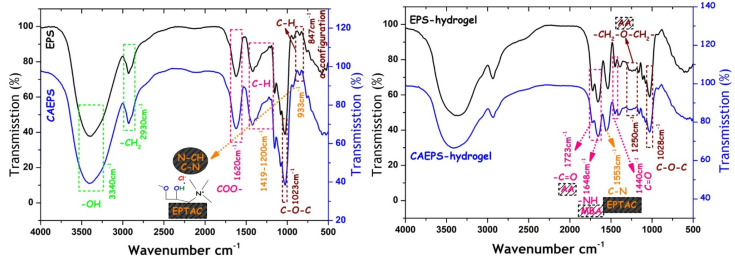
FT-IR spectra of EPS, CAEPS, EPS-, and CAEPS-hydrogel.

In this research, the functional groups of EPS and EPS synthesized samples were characterized by FT-IR. In Figure 3, the infrared spectrum of EPS was similar to CAEPS, which showed that the peak of -OH stretching vibration and -CH_2_ stretching vibration occurred at 3340 cm^−1^ and 2930 cm^−1^, respectively. The absorption peak of COO– and C–O–C appeared at 1620 cm^−1^ and 1023 cm^−1^, respectively. The stretching vibration of C–N at 933 cm^−1^ was increased significantly in CAEPS, which was related to the C–N in EPTAC. In the FT-IR curve of hydrogel, the stretching vibration of –OH (3430 cm^−1^), –S=O (1440 cm^−1^), –C=O (1440 cm^−1^), and –C–O–C (1028 cm^−1^) were ascribed to basic properties of polysaccharides and carbasugars. Moreover, several new peaks were determined, the peaks at 1723 cm^−1^, 1648 cm^−1^, and 1250 cm^−1^ were ascribed to the stretching vibration peak of –C=O in AA, –NH in NMBA, and –CH_2_–O–CH_2_ in AA, respectively. In addition, a peak at 1553 cm^−1^ of CAEPS-hydrogel was ascribed to the stretching vibration of C–N in EPTAC. In conclusion, EPS and CAEPS were cross-linked with NMBA as hydrogels, respectively, which retained a variety of active groups with adsorption potential.

#### 2.1.4. TG

**Figure 4:**
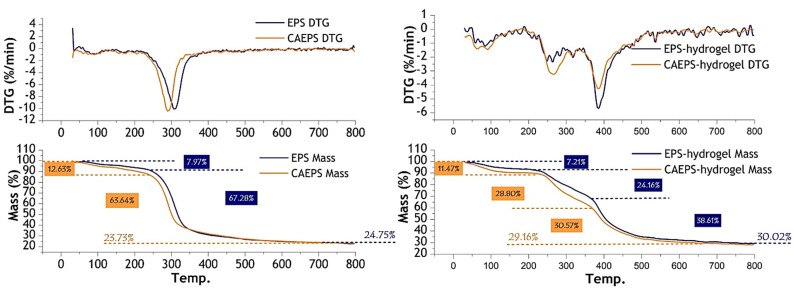
Thermogravimetric analysis curve of EPS, CAEPS, EPS-, and CAEPS-hydrogel. Note: 3 mg EPS and CAEPS was measured for room temperature to 800 °C in the atmosphere of nitrogen, and the heating rate was 20 °C/min and the gas flow rate was 70 mL/min.

The thermal stability of the EPS, CAEPS, EPS-, and CAEPS-hydrogels were determined by TG/DT thermal analysis. It can be seen from Figure 1 that the decomposition temperatures of EPS and CAEPS were 311 °C and 287 °C, respectively, which showed that the thermal stability of EPS was slightly better than CAEPS. From Figure 4, it can be found that the TGA curves of EPS- and CAEPS-hydrogels were divided into three stages. In the first stage, EPS- and CAEPS-hydrogels lost weight by 7.21% and 11.47%, respectively, at around 80 °C–100 °C, which was caused by the loss of free water and kinds-bulk water. That also showed that the moisture content of CAEPS-hydrogels was higher than EPS-hydrogels. The second stage was the thermal decomposition of EPS- and CAEPS-hydrogels reticular structure; at the start, the thermal decompositions of EPS- and CAEPS-hydrogels were similar, and decreased 24.16% and 28.80% at ~265 °C. In the third stage, EPS- and CAEPS-hydrogels were further thermally decomposed and carbonized, both of which thermally decomposed at around 385 °C, and decreased 38.61% and 30.57%, respectively. The final residual mass fractions of EPS- and CAEPS-hydrogels were 30.02% and 29.16%, respectively. The results above indicated that the thermal stability of EPS- and CAEPS-hydrogels was consistent, and the weight loss percentage of the three stages was slightly different when the final mass fraction was similar, which indicated that both EPS- and CAEPS-hydrogels were high cross-linking density, and the thermal stability of CAEPS and CAEPS-hydrogels were not affected by the cationic modification.

#### 2.1.5. Morphological Observations

**Figure 5:**
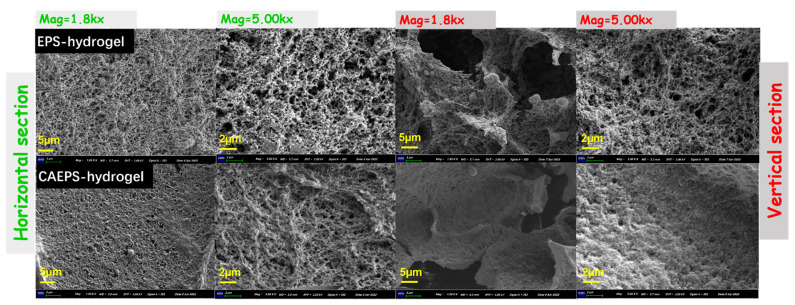
SEM image of EPS- and CAEPS-hydrogel at 1.8K× Magnification, scale bar: 5 µm; 5.00K× Magnification, scale bar: 2 µm, respectively. Note: The samples were subjected to vacuum freeze-drying to remove moisture and tested at room temperature.

From a theoretical perspective, the EPS- and CAEPS-hydrogels with a three-dimensional network structure will be produced after the monomer was crosslinked. As can be seen from the photographs of EPS- and CAEPS-hydrogels (Figure 5), EPS- and CAEPS-hydrogels have highly developed light three-dimensional structure of honeycomb with translucent and thin walls accompanied by a large number of folds. The EPS- and CAEPS-hydrogel was rich in pores of all sizes and had a relatively smooth surface with some gully cracks. The pore structure on the surface is caused by the loss of moisture of the hydrogel during vacuum drying.

**Figure 6:**
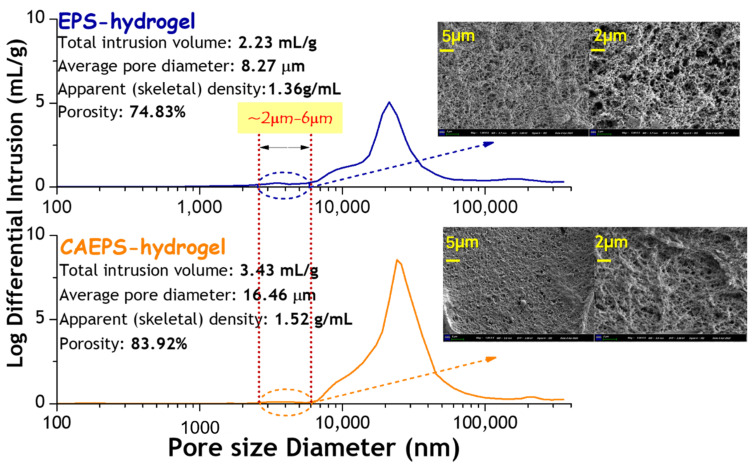
Pore size distribution of EPS- and CAEPS-hydrogels.

As shown in Figure 5, the stagger-distributed honeycomb holes were distributed in EPS- and CAEPS-hydrogel, which provided abundant water channels for the hydrogel to adsorb pollutants in the water environment. Compared with EPS, at the 1.8K× and 5.00K× magnification of the horizontal section, the surface pore of CAEPS-hydrogel was smaller and the crosslinking structure was more compact, which was also reflected at the same magnification of the vertical section. This indicated that, within a certain pore size scale range (~1–5 µm), the pores distributed of CAEPs-hydrogel were fewer and the gel skeletal was denser, which was also verified in the pore size diameter analysis of EPA/CAEPS-hydrogels (Figure 6). Meanwhile, the horizontal and vertical section of the two gels were significantly different, the main distinction was uneven distribution of large size holes in vertical section and the holes of horizontal section were uniform in distribution and smaller in size. This was due to the fact that, during freezing, the moisture in the gel formed ice crystal at low temperature. With the transfer of heat, the ice peak gradually approaches the surface of the gel and formed the columnar structure in the vertical direction. Therefore, the large holes appeared in the vertical section.

Furthermore, the element distribution of EPS- and CAEPS-hydrogel was elucidated by SEM-EDS mapping, from which the differences in crosslinking reactions were speculated. As shown in Figure S1, there was little difference in the content of C between CAEPS- and EPS-hydrogel, which might be due to the similar number of active sites for substitution and cross-linking of carbohydrate chains. The source of element Cl in CAEPS-hydrogel was EPTAC (C_6_H_14_ClNO, C:Cl = 6:1) and ECH (C_3_H_5_ClO, C:Cl = 3:1) and the source of Cl in EPS was ECH (C_3_H_5_ClO, C:Cl = 3:1). The contents of Cl in CAEPS-hydrogel were significantly lower than that in EPS, and the former was about 0.5 times the latter which indicated that the degree of crosslinking between CAEPS and ECH was significantly lower than EPS. The sources of N element in CAEPS-hydrogel were EPTAC (C:N = 6:1) and NMBA (C_7_H_10_N_2_O_2_, C:N = 3.5:1), and the source of N element in EPS was NMBA (C:N = 3.5:1). With a similar amount of carbon, the content of N in CAEPS-hydrogel was significantly higher than that in EPS-hydrogel, the former was about 1.76 times of the latter which indicated that CAEPS has a significantly higher cross-linking degree with NMBA than EPS. The content of the O element in EPS-hydrogel was slightly higher than that in CAEPS-hydrogel, which may be caused by, in EPS, more active sites for cross-linking with ECH (C:O = 3:1) and, when in CAEPS, more active sites for cross-linking with NMBA (C:O = 3.5:1), and also some active sites in CAEPS were replaced by EPTAC (C:O = 6:1). This result was consistent with the conclusion of Cl element analysis. In conclusion, in the two-step crosslinking reaction, EPS and ECH were more deeply crosslinked, while CAEPS was more deeply crosslinked with NMBA.

#### 2.1.6. Pore Size Distribution Analysis of EPS- and CAEPS-Hydrogel

As shown in Figure 6, the pore size distribution curves of the two hydrogels both showed a single peak, which indicated a uniform pore size distribution. The average pore diameter of CAEPS-hydrogel was significantly larger than that of EPS-hydrogel, which were 16.46 µm and 8.27 µm, respectively. The total intrusion volume of EPS- and CAEPS-hydrogel were 2.23 mL/g and 3.43 mL/g, respectively. The porosity of EPS- and CAEPS-hydrogel were 74.83% and 83.92%, respectively. Compared with EPS-hydrogel, the more developed pore distribution of CAEPS-hydrogel provided and exposes more active sites to bind to contamination factors, and provided more water channels for substance exchange with the external water environment. Compared with EPS-hydrogel, the apparent (skeletal) density of CAEPS-hydrogel was greater, which indicated that CAEPS-hydrogel had a frame with tighter texture. It can be seen from the pore distribution curve that pores the size of EPS-hydrogel were distributed in the range of 2–6 µm, while there were almost no pores in the same range in CAEPS-hydrogel. This conclusion is consistent with the results presented by SEM images.

#### 2.1.7. Surface Wettability of EPS-Xerogels and CAEPS-Xerogels

**Figure 7:**
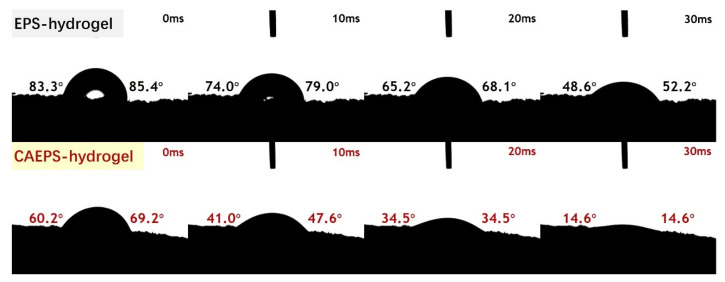
The water droplet on EPS- and CAEPS-hydrogel and the WCA of EPS- and CAEPS-hydrogel, respectively. Note: The water droplet is 2 µL.

The hydrophilia of the EPS- and CAEPS-xerogels were evaluated via contact angle measurement. As can be seen from Figure 7, the xerogels prepared from EPS- and CAEPS-hydrogels had good hydrophilic properties. Furthermore, compared to EPS-xerogels, the contact angle of CAEPS-xerogels was smaller at the same time.

According to Figure 6, at the 180× and 500× magnification, significantly larger pores were more unevenly distributed on the surface of the CAEPS-hydrogel. This indicated that the surface of CAEPS-hydrogel was rougher than that of EPS-hydrogel., which may lead to better surface wettability of CAEPS. In addition, in the analysis results of FT-IR (Section 2.1.3), the active group-quaternary ammonium in CAEPS was retained in CAEPS-hydrogels, which might lead to the better hydrophilic properties of CAEPS-hydrogels.

### 2.2. Swelling Behaviors of the EPS- and CAEPS-Hydrogels

**Figure 8:**
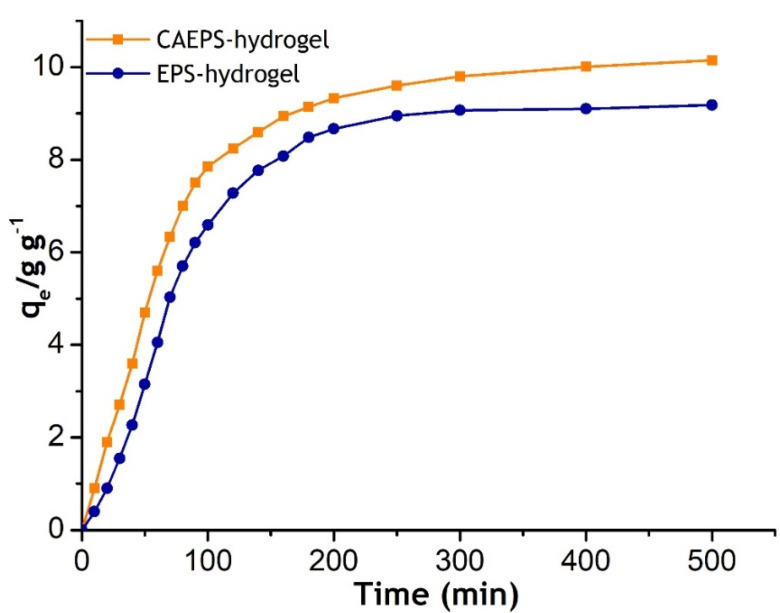
Swelling kinetic curve of EPS-hydrogel and CAEPS-hydrogel. Note: The freeze-dried gels were placed in distilled water for static absorption.

In the application field, the swelling property of the hydrogels was an important index of hydrogels. Liquid–solid contact time was one of the important parameters for water absorption and swelling performance of hydrogel application. As shown in Figure 8, the water absorption rate of EPS- and CAEPS-hydrogels increased rapidly in 150 min and reached saturation after 200 min. The swelling process of EPS- and CAEPS-hydrogel could be divided into two stages: fast stage and slow stage. Initially, the water absorption rate was very fast because of the large number of binding sites, which can bind to water rapidly. In the slow stage, the fewer binding sites lead to a low adsorption rate and, finally, saturation. Compared with EPS, the CAEPS water absorption rate was higher, which was related to the better hydrophilicity of CAEPS that was consistent with the conclusion of the surface wettability test analysis. Furthermore, the water absorption capacity of CAEPS-hydrogel was better than EPS-hydrogel, which was related to the developed pore structure, higher porosity, and total intrusion volume.

### 2.3. Adsorption Properties of EPS- and CAEPS-Hydrogels on Methylene Blue, Cefuroxime, and Cr (VI) (Na_2_Cr_2_O_7_)

**Figure 9:**
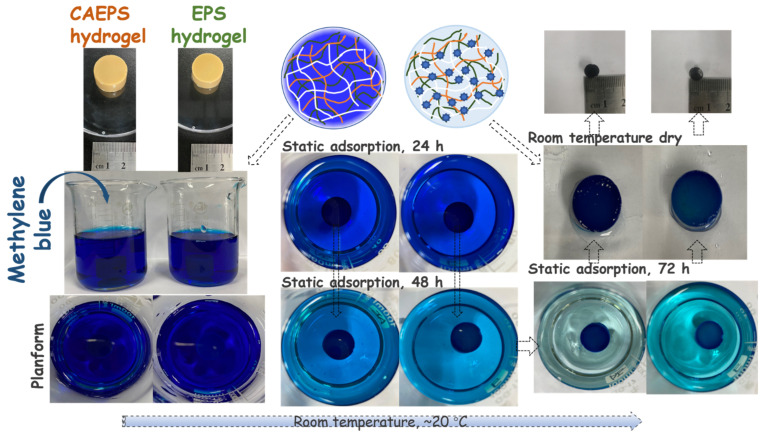
The static adsorption schematic diagram of EPS- and CAEPS-hydrogel on methylene blue.

**Figure 10:**
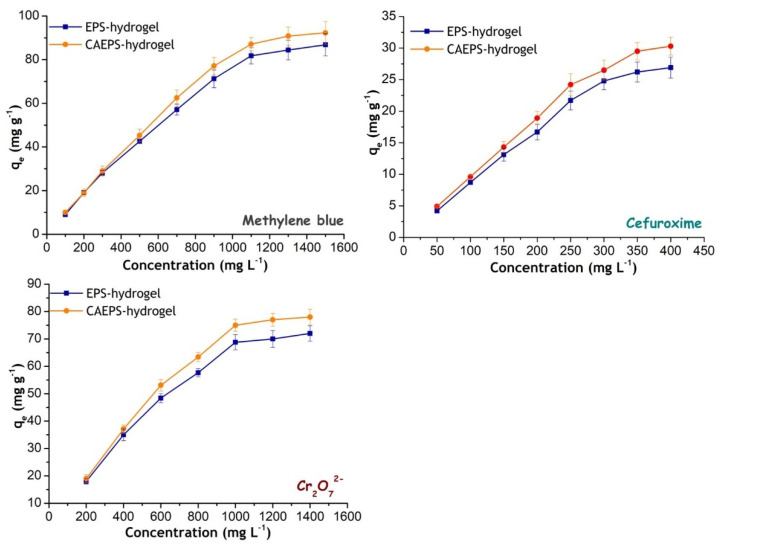
Effect of methylene blue, Cefuroxim, and Cr_2_O_7_^2−^ concentration on the adsorption of EPS- and CAEPS-hydrogel and the isotherm model, respectively. Note: About 10 g of wet EPS- and CAEPS-hydrogel was added to the prepared 50 mL in different concentrations of MB solution for 72 h, Cefuroxime solution, and K_2_Cr_2_O_7_ solution for 48 h until static adsorption equilibrium in room temperature (~20 °C) was reached, respectively.

The prepared hydrogel was immersed in the solution containing pollutants factors (MB, Cefuroxime, and Cr (VI)) and placed at room temperature for static adsorption. After 72 h, the gel was removed, and the solution was measured for the content of the pollutants factor. The gel adsorption process was shown in Figure 9, more MB pigment was adsorbed on the surface of CAEPS-hydrogel with a more obvious dark blue color, while the remaining MB in the solution was significantly less, indicating that CAEPS-hydrogel might have better adsorption performance. Meanwhile, in can be seen in Figure 10 that the adsorption capacity of EPS- and CAEPS-hydrogel increased gradually with the increase of the initial concentration of methylene blue, cefuroxime, and Cr (VI) until the final adsorption capacity of the hydrogel tends to be stable, which was due to the fixed amount of hydrogel with the certain number of adsorption sites, and the increased of pollution factor concentration led to fewer adsorption sites. Finally, the adsorption capacity of the pollution factor reached saturation. To investigate the adsorption mechanism, the relationship between adsorption capacity and pollution factor concentration was analyzed through the adsorption isotherms model.

The Langmuir adsorption isotherm model [39] assumed that adsorption was monolayer adsorption, which had no saturated atomic force field on its surface. The field would be saturated when the adsorbent surface was covered by a layer of adsorbent molecules [11].

The non-linear Langmuir adsorption isotherm equation was as follows:

(1) $$q_{e}=\frac{q_{m,L}\times K_{L}\times C_{e}}{1+K_{L}\times C_{e}}$$

where *C_e_* (mg L^−1^) was the concentration of adsorbate at equilibrium, *q_e_* (mg g^−1^) was the adsorption capacity of adsorbent at equilibrium, *K_L_* (L mg^−1^) was the Langmuir adsorption constant, and *q_m,L_* (mg g^−1^) was the theoretical maximum adsorption capacity.

The non-linear Freundlich adsorption isotherm model [40] assumed that the adsorbent surface was heterogeneous, the adsorbate adsorption was multi-layered, and there was interaction between adsorbents.

(2) $$q_{e}=K_{F}\times C_{e}{}^{\frac{1}{n}\times C_{e}}$$

where *C_e_* (mg L^−1^) was the concentration of adsorbate at equilibrium, *q_e_* (mg g^−1^) was the adsorption capacity at equilibrium, *K_F_* (L g^−1^) was the adsorption constant related to adsorption capacity, and *n* was the adsorption constant related to surface inhomogeneity.

As shown in Figure 10, the corresponding isotherm model parameters were obtained by fitting. It was clear from Table 1 that the process of EPS- and CAEPS-hydrogel adsorption of MB, Cefuroxime, and Cr (VI) were more consistent with the fitting of nonlinear-Langmuir than nonlinear-Freundlich, of which the correlation coefficient R^2^ were all above 0.95, which were better than the correlation coefficient of nonlinear-Freundlich isotherm models. This showed that the process of adsorption of MB, Cefuroxime, and Cr (VI) by EPS- and CAEPS-hydrogel was mainly by the chemical adsorption of the single molecular layer of Langmuir. Combined with the parameters 1*/n* in the Freundlich model, it could be inferred that the adsorption processes were more likely to occur. Based on the fitting results of the isotherm model, it was concluded that the whole adsorption process was mainly endothermic chemical adsorption with a single molecular layer and spontaneous reaction. Compared with EPS-hydrogel, the adsorption rate and saturated adsorption capacity of CAEPS-hydrogel for MB were both higher than EPS-hydrogel, which indicated that the positive charge in CAEPS-hydrogel did not significantly inhibit the binding between active sites and MB with the same charge, higher adsorption rate might be due to its excellent hydrophilicity of CAEPS-hydrogel. In the adsorption performance experiment, the pH value of the Cefuroxim/deionized water solution at low concentration (50 mg L^−1^) was about 6.83, and with the increase of Cefuroxim concentration to 400 mg L^−1^, the pH value of the solution decreases to about 6.35. According to the pKa = 2.5 of Cefuroxim, it could be speculated that the –COOH dissociation of Cefuroxim was greater than -NH_2_ in deionized water, which lead to electronegativity of Cefuroxim. Meanwhile, the adsorption rates of CAEPS-hydrogel for Cefuroxim and Cr (VI) were higher, which may be due to the partly active sites of CAEPS-hydrogel contained quaternary ammonium groups, which had a positive charge and tends to combine with the Cefuroxim and Cr(VI) with the negative charge. The saturated adsorption capacity of CAEPS-hydrogel was higher, which might be due to the higher porosity that exposed more adsorption sites to combine with pollution factors. These conclusions indicated that cationic modification of EPS-polysaccharides could significantly improve the adsorption rate and saturation adsorption capacity of polysaccharides-based hydrogel to electronegative pollution factors.

## 3. Conclusions

The wide application of hydrogel prompted us to prepare a natural composite hydrogel based on Enteromorpha, an algal pollutant from the Qingdao coast. The *Entermorpha prolifera* polysaccharide-based hydrogel EPS- and CAEPS-hydrogel with excellent adsorption performance was successfully prepared by a double network hydrogel polymerization method. In this study, EPS were extracted from *Enteromorpha prolifera* and cationic EPS (CAEPS) with quaternary ammonium group were prepared. Hydrogels for pollutant adsorption were prepared based on the two EPS polysaccharides. The structure of EPS- and CAEPS-hydrogel was characterized by AFM, FT-IR, TGA, SEM-EDS, and MIP. The analysis of AFM, FT-IR, and TGA showed that cationic modification was beneficial to the expansion and dispersion of polysaccharide main chain in water, but also slightly affected the thermal stability. Furthermore, the analysis of TGA, SEM-EDS, and MIP showed that the hydrogels synthesized based on EPS and CAEPS had developed pore distribution and honeycomb skeleton, and there was no significant difference in thermal stability between the two. In addition, the CAEPS-based hydrogels had more uniform pore distribution, higher-density gel skeleton, and higher porosity than EPS-based hydrogels. In terms of performance, according to WCA analysis, CAEPS-hydrogel has better hydrophilicity, higher water absorption rate, and higher saturated water absorption than EPS-hydrogel. In the study of gel adsorption performance, the two gels were used to adsorb the three types of pollution factors: MB, cefuroxime, and Cr (VI). The adsorption experiment results showed that the adsorption of the two hydrogels on the three pollution factors was consistent with the Langmuir isotherm model, which proved that the adsorption process was monolayer adsorption. According to the adsorption isotherm, cationic modification can improve the adsorption performance of hydrogel for MB, cefuroxime, and Cr (VI). The maximum adsorption of CAEPS- and EPS-hydrogel on Cr^6+^ (83.67 mg g^−1^, 80.82 mg g^−1^), which were similar to hydrogels, have been studied, such as Cellulosic biopolymer (76 mg g^−1^) [41], PE-PD/GO (87 mg g^−1^) [42], and M-CS-AnGS (83 mg g^−1^) [43]. At present, the enhancement of adsorption function of hydrogel was studied. Zeng et al. [25] prepared a pullulan/polydopamine-hydrogel, of which the maximum adsorption of Cu^2+^ reached 100.9 mg g^−1^. Facchi et al. [23] studied the adsorption capacity of chitosan/alginate-hydrogel with Fe_3_O_4_@SiO_2_ added to reached 220 mg L^−1^ on Pb^2+^. Wang et al. [44] developed an adsorbent composed of MOFs and cellulose nanocrystals, which displayed the excellent maximum adsorption ability of 558.7 mg g^−1^. The adsorption capacity of hydrogels above-mentioned was significantly stronger than that of *Enteromorpha prolifera* polysaccharide-based hydrogel of this study, which also suggests that the enhancement of EPS-hydrogel adsorption could be further studied in the future. The preparation of hydrogels by *Enteromorpha prolifera* polysaccharide was aimed to realize the resource utilization of pollutants, of which the performance also can be optimized through cationic modification. Therefore, the research strategy and conclusions were instructive and practical.

## 4. Experiments

### 4.1. Experimental Materials

Epoxy chloropropane (ECH), acrylic acid (AA), and N’N-methylene bisacrylamide (NMBA) were bought from McLean Biochemical Co., Ltd. (Shanghai, China). Sodium hydroxide and potassium peroxodisulfate (KPS) were from Compare Chemical Reagent Co., Ltd. (Shanghai, China). Potassium dichromate (K_2_Cr_2_O_7_) was from Aladdin Chemical Reagent Co., Ltd. (Shanghai, China). Cefuroxime (99%) was from McLean Biochemical Co., Ltd. (Shanghai, China). Methylene blue (MB), ethanol, and other experimental reagents were bought from Concord Chemical Reagents Co., Ltd. (Tianjin, China). All aqueous solutions used for reactant polymerization, water swelling, and adsorption studies of hydrogels were prepared using deionized water.

### 4.2. Extraction of Enteromorpha Prolifera Polysaccharide (EPS)

Fresh *Enteromorpha prolifera* (EP) was bought from HAITAI biological group Co., LTD (Qingdao, China). A certain amount of fresh EP was placed outdoor with sufficient sunlight and good ventilation for natural drying, after intensive drying the EP was crushed by a high-speed grinder and screened through 80 mesh to obtain even and dried EP powder. Crude EP-polysaccharides (EPS) were extracted by the hot water extraction method [45]. The EP powder was immersed and homogeneous mixed in 20 times the weight of water, and the mixed liquor was stirred for 2 h in a water bath at 60 °C. After extraction, the mixture was centrifuged at 8500 rpm to separate the supernatant. The polysaccharides in the supernatant were collected by the fractional alcohol precipitation method. Specifically, an equal volume (1:1) of anhydrous ethanol was added to the supernatant to the liquid with an ethanol concentration of 50%, and the mixture was placed at 4 °C for 12 h, then the mixture was centrifuged at 8500 rpm and the precipitation (EPS) was obtained. It was noticeable that the purpose of using an equal ratio of ethanol to precipitate polysaccharides was to obtain the components with the larger molecular weight in crude EPS.

### 4.3. Preparation of Cationic Entermorpha Polysaccharide and Cationic Entermorpha Polysaccharide Hydrogel (EPS- and CAEPS-Hydrogel)

A certain amount of NaOH was put into a 50 mL beaker, and it was dissolved with a certain amount of deionized water. After cooling to room temperature, a certain amount of CHPTAC was added to the mixture. After the mixture was thoroughly shaken and mixed, it was placed in the environment of 4 °C for 10 min to convert CHPTAC into EPTAC under alkaline conditions.

The EPS- and CAEPS-hydrogel were synthesized according to the following steps [15], a certain amount of NaOH was dissolved in distilled water to prepare a 3 mol/L NaOH solution, and 0.2 g mL^−1^ EPS and CAEPS solution (5 mL) was adjusted pH to 11 after stirred for 15 min, the polysaccharide solution was added with 500 µL ECH and reacted in 65 °C for 5 h. Then 0.2 g AA, 0.3 g NMBA, and 0.02 g KPS were premixed fully and then added to the EPS solution. The EPS solution was intensively mixed by ultrasonic cleaner for 5 min and the system was maintained at 70 °C for 2 h. Finally, the synthesized hydrogel was washed with distilled water and anhydrous ethanol, respectively, to remove the unreacted component. The hydrogel could be kept in distilled water at 4 °C for short-term storage and the dry gel was prepared by freeze-dried at −40 °C for 48 h.

### 4.4. Experiment and Reaction Mechanism

**Figure 11:**
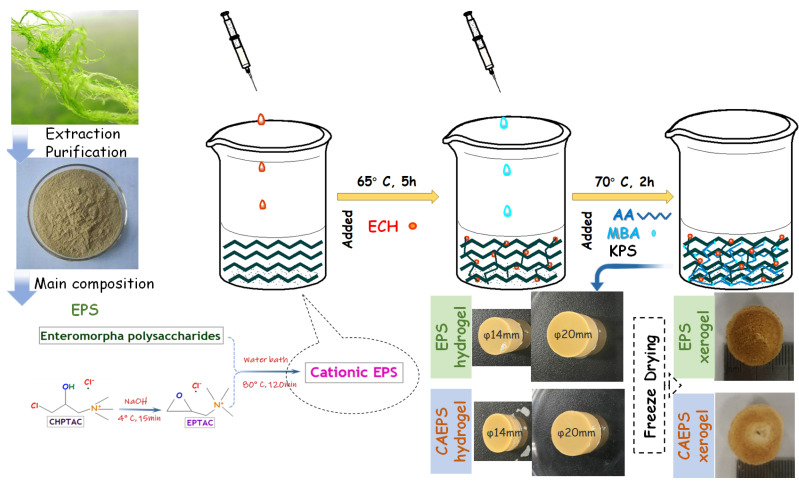
The fabrication process of the EPS and CAEPS-based double network hydrogel.

The EPS with multiple hydroxyl groups and the O–H bond was broken easily under the action of heat and initiator. In this research, cationic modification of EPS was achieved through etherification reaction, that is, under alkaline conditions, the partial hydroxyl group in EPS-chain was replaced by the quaternary ammonium group from the EPTAC, which gave EPS a certain amount of cationic charge. Double-crosslinked polysaccharide hydrogel was prepared by a two-step cross-linking method. Firstly, under alkaline conditions, the ring-opening of the epoxy bond and the fracture of the C–Cl bond were partially cross-linked with EPS; thus, a hydrogel with a single network structure was formed. Meanwhile, there were still a large number of uncross-linked active hydroxyl groups and some free single chains in EPS. Secondly, under the action of initiating agent (KPS), the active hydroxyl received the attack of AA and produced a large number of polymers branched chains, and the second reticulated architecture was formed. All the grafted chains were cross-linked together to form reticulated architecture macromolecules under the action of cross-linking agent NMBA. The free monomer and single-chain were removed by anhydrous ethanol, and finally, the hydrogel samples were obtained by freeze-drying. The specific processes of the above-mentioned reaction are shown in Figure 11.

### 4.5. Characterization of EPS, CAEPS, EPS-, and CAEPS-Hydrogel

#### 4.5.1. Monosaccharide Composition Analysis of EPS

The uronic acid contents of samples were determined by HPLC [46]. Twelve monosaccharides standard 50 µg per type (mannose, ribose, rhamnose, glucuronic acid, galacturonic acid, N-acetyl-glucosamine, glucose, N-acetyl-galactose, galactose, xylose, arabinose, fucose) were dissolved into 1 mL deionized water. A volume of 250 µL standard solution above was mixed with 250 µL NaOH (0.6 M) and 500 µL PMP-methanol (0.4 M) at 70 °C for 1 h, and 500 µL HCl (0.3 M) was added to adjusting the pH. After the reaction, the mixture was extracted by 1 mL chloroform three times, the extracted supernatant was collected for analysis by HPLC (LC-20AD, Shimadzu, Kyoto, Japan). The sample EPS (15 mg) was hydrolyzed with 10 mL of 2 M TFA at 120 °C for 4 h. After that, 1 mL of hydrolysate and methanol (10 mL) was added to the ampoule was dried by a termovap sample concentrator to get rid of the residual and the step above was repeated three times, and 1 mL of deionized water was added to redissolve. The derivatization steps of samples refer to the monosaccharide standard above. The mixture was analyzed by the HPLC equipped with an Xtimate C18 column (4.6 mm × 200 mm × 5 µm) and an UVD. The mobile phase was acetonitrile-0.05 M phosphate buffer (17:83), the flow rate was 1.0 mL/min, and the determined wavelength was 250 nm.

#### 4.5.2. AFM Analysis

The sample powder of EPS and CAEPS were prepared into a solution with a concentration of 0.1 µg mL^−1^, and 5 µL of the solution was uniformly coated on the surface of the mica sheet, which was left and dried at room temperature, and then the dried mica sheet was placed on the sample table for detection by AFM SPA400 (SEIKO, Tokyo, Japan).

#### 4.5.3. FT-IR Analysis

The functional group and characteristic bonding were measured [47] by Nicolet iN10 FT-IR spectrometer (Thermo Fisher, Waltham, MA, USA), within the wavenumber range of 4000–400 cm^−1^ by potassium bromide pellet technique.

#### 4.5.4. TG Analysis

To investigate the thermal stability, EPS, CAEPS, EPS-, and CAEPS-hydrogel powders were determined by TG analysis on a thermal analyzer (STA 449F3, Netzsch, Germany). About 3 mg of EPS and CAEPS was placed on the sample tray and heated from room temperature to 800 °C in the atmosphere of nitrogen. The heating rate was 20 °C/min and the gas flow rate was 70 mL min^−1^, respectively.

#### 4.5.5. SEM-EDS Analysis

The appropriate amount of samples were placed on the metallic sample stage and coated with gold. The microstructure of the samples was analyzed through a field-emission scanning electron microscopy EDS (OxfordX-MAX) attached to the SEM (ZEISS, sigma300, Jena, Germany).

#### 4.5.6. Contact Angle Measurement

The water contact angle (WCA) was measured by a contact angle meter (Dataphysics OCA20, Filderstadt, Germany) with a water droplet of 2 µL.

#### 4.5.7. Mercury Intrusion Analysis of Pore Size Distribution

The average pore diameter, total intrusion, and porosity of EPS- and CAEPS-hydrogel were measured by MIP through AutoPore IV 9500 (Micromeritics, Norcross, GA, USA).

### 4.6. Hydrogel Swelling Capacity

The swelling capacity of EPS- and CAEPS-hydrogel was measured according to previous research work [11]. At 25 °C, the dry EPS- and CAEPS-hydrogel was weighed as *m*1 g and put into deionized water until the hydrogel reached adsorption equilibrium. Then the mass of the adsorbed hydrogel was weighed as *m*2 g. The swelling rate of EPS- and CAEPS-hydrogel was calculated by the following formula:

(3) $$q_{e}\;=\;\frac{m2\;-\;m1}{m1}$$

where *q_e_* (g g^−1^) is the quality of adsorbed water per unit mass of hydrogel. The data obtained in this paper are averaged by multiple measurements.

### 4.7. Adsorption Properties of Methylene Blue, Cefuroxim, and Cr (VI) onto EPS- and CAEPS-Hydrogel

The adsorption capacity of EPS- and CAEPS- hydrogel was measured according to previous research work [8]. The diffusion study was carried out using nonlinear isotherm models (Langmuir, Freundlich) to analyze the adsorption characteristics [48]. About 10 g of wet EPS- and CAEPS-hydrogel was added to the prepared 50 mL in different concentrations of MB solution, Cefuroxime solution, and K_2_Cr_2_O_7_ solution, respectively. After static adsorption equilibrium reached temperature for 48 h of Cefuroxime and K_2_Cr_2_O_7_, 72 h of MB (~20 °C). The hydrogel adsorption capacity was calculated by the following equation:

(4) $$q_{e}=\frac{\left(C_{0}-C_{e}\right)V}{m}$$

where *C*_0_ (mg L^−1^) and *Ce* (mg L^−1^) represent the initial concentration and the equilibrium concentration of MB, cefuroxime, and K_2_Cr_2_O_7_ in the solution, respectively. *q_e_* (mg g^−1^) is the adsorption capacity of sample hydrogel; *V* (L) is the volume of MB, cefuroxime, and K_2_Cr_2_O_7_ solution; and *m* (g) is the weight of adsorbent. It is noticeable that the residual MB concentration was measured by ultraviolet spectrophotometer (HACH, Loveland, CO, USA) at 663 nm, the residual Cefuroxim concentration was measured by ultraviolet spectrophotometer at 273 nm, and the residual Cr^6+^ concentration in the solution was determined using an inductively coupled plasma emission spectrometer (ICP-OES, Agilent, Santa Clara, CA, USA).

## Figures and Tables

**Table 1:** Adsorption isotherm parameters.

Adsorption	MB		Cefuroxim		Cr_2_O_7_^2−^	
	EPS-Hydrogel	CAEPS-Hydrogel	EPS-Hydrogel	CAEPS-Hydrogel	EPS-Hydrogel	CAEPS-Hydrogel
Nonlinear-Langmuir						
*q_m,L_* (mg g^−1^)	95.28	96.41	33.92	35.78	80.82	83.67
*K_L_* (L mg^−1^)	0.0146	0.008	0.0389	0.1056	0.0139	0.0263
R^2^	0.9690	0.9798	0.9883	0.9837	0.9867	0.9853
Nonlinear-Freundlich						
*K_F_* ((mg g^−1^) (L mg^−1^)^1/*n*^)	11.06	6.6152	4.14	8.29	9.21	14.25
1/*n*	0.3328	0.3954	0.4086	0.3174	0.3349	0.2865
R^2^	0.9639	0.9188	0.9053	0.8767	0.9378	0.9

## Supplementary Materials

The following supporting information can be downloaded at: https://www.mdpi.com/article/10.3390/gels8090546/s1, Figure S1: SEM-EDS mapping of EPS/CAEPS-hydrogel, EDS spectrum of EPS/CAEPS-hydrogel and the middle of EDS panels are the main element content of these substrates.

## Abbreviations

EP: *Entermorpha prolifera*; EPS*: Entermorpha prolifera* polysaccharides; CAEPS: Cation *Entermorpha prolifera* polysaccharides; HPLC: High performance liquid chromatography, AFM: Atomic force microscope, FT-IR: Fourier transform infrared, TGA: Thermo gravimetric analysis, SEM-EDS: Scanning electron microscopy and energy spectrum, WCA: Water contact angle; MB: Methylene blue; ECH: Epoxy chloropropane, AA: acrylic acid, NMBA: N’N-methylene bisacrylamide; KPS: Sodium hydroxide and potassium peroxodisulfate; K2Cr2O7: Potassium dichromate; CHPTAC: 3-chloro-2-hydroxypropyl trimethyl ammonium chloride solution, EPTAC: 2, 3-epoxy-propyl trimethyl ammonium chloride, PMP: Propylene glycol methyl ether propionate, HPLC: High Performance Liquid Chromatography, TFA: Trifluoroacetic acid, UVD: Ultraviolet detector, MIP: Mercury intrusion porosimetry, PMP-methanol: Propylene glycol methyl ether propionate methanol.
