# Supplementary material for: Structure and Adsorption Performance of Cationic Entermorpha prolifera Polysaccharide-Based Hydrogel for Typical Pollutants: Methylene Blue, Cefuroxime, and Cr (VI)
Source: Gels. 2022 Aug 29;8(9):546. doi: 10.3390/gels8090546 (PMC9498468; doi:10.3390/gels8090546)
Supplement: Supplementary file 1 [file gels-08-00546-s001.zip › gels-1783421-supplementary.pdf]

gels

MDPI

Article

Structure and Adsorption Performance of Cationic *Entermorpha prolifera* Polysaccharide-Based Hydrogel for Typical Pollutants: Methylene Blue, Cefuroxime, and Cr (VI)

Xiaolei Ma [1,*], Duomo Duan [2], Jinbin Chen [1] and Baolong Xie [1,*]

[1] The Institute of Seawater Desalination and Multipurpose Utilization, SOA, Tianjin 300192, China
[2] Tianjin Rehabilitation Center, The PLA Joint Logistic Support Force, Tianjin 300191, China
* Correspondence: huandaoyu@126.com (X.M.); xiebaolong@tju.edu.cn (B.X.)

Supplementary Materials

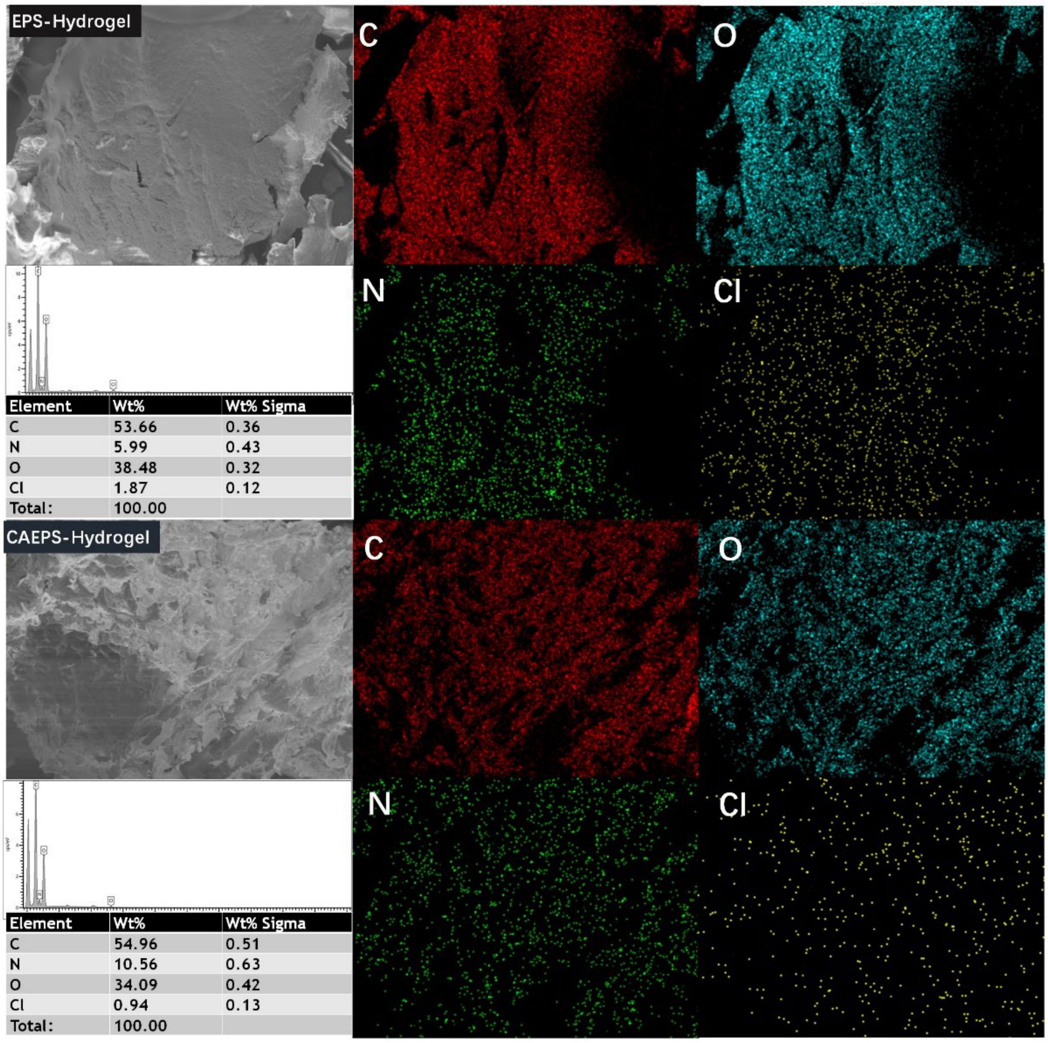

Figure S1. SEM-EDS mapping of EPS/CAEPS-hydrogel, EDS spectrum of EPS/CAEPS-hydrogel and the middle of EDS panels are the main element content of these substrates.
